# Supplementary material for: Old Drugs with New Tricks: Efficacy of Fluoroquinolones to Suppress Replication of Flaviviruses
Source: Viruses. 2020 Sep 13;12(9):1022. doi: 10.3390/v12091022 (PMC7551155; doi:10.3390/v12091022)
Supplement: Supplementary file 1 [file viruses-12-01022-s001.zip › Scroggs_Revision_Supplementary/Scroggs_Suppl_Tables.docx]

**Table S1.** ANOVA results and pairwise t-test comparisons for ZIKV titer after treatment with enoxacin, difloxacin and ciprofloxacin from the time-of-addition assays in Fig. 2.

|  | Enoxacin | | Difloxacin | | Ciprofloxacin | |
| --- | --- | --- | --- | --- | --- | --- |
| Concentration (μM) | 18.1 | 24.4 | 35.9 | 50.0 | 25.0 | 116.1 |
| MOI | 1.0 | 0.2 | 0.2 | 1.0 | 1.0 | 0.2 |
| F (df), P | 2.6 (8, 18),  0.04 | 3.6, (8,18),  0.01 | 2.8 (8, 18), 0.04 | 12.8 (8, 18), 5.2e-6 | 16.2 (8, 18), 2.3E-7 | 14.7 (8, 18), 1.9e-6 |
| Time of addition (hour p.i.) |  | |  | |  | |
| Media | AB | AB | A | A | A | A |
| -2 | AB | AB | AB | BC | CD | A |
| 0 | A | ABC | AB | CD | BC | A |
| 2 | BC | CD | B | CD | E | B |
| 4 | BC | D | AB | D | E | B |
| 6 | C | CD | B | D | DE | B |
| 8 | ABC | BCD | B | CD | DE | B |
| 12 | ABC | AB | B | A | BC | A |
| 18 | A | A | B | AB | B | A |

**Table S2**. Pairwise t-test comparisons of mean percent weight change from *in vivo* ZIKV infection in Fig 3a

| Virus + Treatment | Day 0 | Day 1 | Day 2 | Day 3 | Day 4 | Day 5 | Day 6 |
| --- | --- | --- | --- | --- | --- | --- | --- |
| Sham + enoxacin | JK | FHI | DEG | BC | BC | B | BC |
| Zika + lactic acid | K | IJ | EFGH | BCDEFGH | BCDEFGH | BCD | A |
| Zika + enoxacin | JK | JK | GHI | DEFGH | EFGHI | CDEF | A |
